# Adapting the Women's empowerment in agriculture index to specific country context: Insights and critiques from fieldwork in India

**DOI:** 10.1016/j.gfs.2019.09.002

**Published:** 2019-12

**Authors:** Soumya Gupta, Vidya Vemireddy, Dhiraj Singh, Prabhu Pingali

**Affiliations:** a Tata- Cornell Institute for Agriculture and Nutrition, Cornell University, United States; b Indian Institute of Management Ahmedabad, India; c Tata- Cornell Institute for Agriculture and Nutrition, India

**Keywords:** Women's empowerment, India, Gender, Agriculture, Indicators

## Abstract

The Women's Empowerment in Agriculture Index (WEAI) is a direct, multi-dimensional measure of women's access to resources and decision-making in various domains of agriculture. However, several challenges characterize its use: adaptation of questionnaires to local agricultural contexts, modifications to index construction once underlying activities and adequacy thresholds are modified, and sensitivity analysis. In this paper, we address such challenges based on our experience of adapting and using the WEAI across 3600 households in India. In doing so we contribute to the methodological and technical base underlying the index, expand the WEAI evidence base for South Asia, and highlight the importance of tailoring the index to specific agricultural contexts in order to impact public policies in a meaningful way.

## Highlights

• The Abbreviated Women’s Empowerment in Agriculture Index (AWEAI) is contextually adapted for 3600 households in India.
• We find that on average women in all four of our locations are disempowered in agriculture.
• Disempowered women lack membership to agriculture-related Self- Help Groups, ownership of land and control over incomes.
• There are significant differences between the original and our India- specific version of the AWEAI in each district.

## 1 Introduction/background

Women’ s empowerment has been measured using multiple indicators. Some of the previously used indicators of women's empowerment include indicators like education (Berti et al., 2004; Smith and Haddad 2000), control over income (Andersen, 2012; Berti et al., 2004; Leroy et al., 2009), gender of household head (Kennedy and Peters, 1992), and control over assets at the time of marriage (Maluccio and Quisumbing, 2003). An example of a multidimensional indicator is the Global Gender Gap Index (GGGI) developed by the World Economic Forum. It measures achievements in four broad outcomes: health, education, economic participation, and political empowerment (World Economic Forum, 2014).

The Women's Empowerment in Agriculture Index (WEAI) was introduced in 2012 as a multidimensional measure to assess women's access to resources and ability to make decisions in five domains of agriculture: 1) Production, 2) Resources, 3) Control over income, 4) Leadership and 5) Time use (Alkire et al., 2013). The WEAI was an improvement over previously used measures of women's empowerment in several ways. For one, it focuses specifically on productive domains in agriculture that become relevant for investigating the role of women's empowerment in the space of agriculture-nutrition linkages. Second, the fact that the index can be disaggregated allows us to identify not just key drivers of women's (and men's) disempowerment, but also the contribution of each of these to overall disempowerment. This can be a useful input when designing context-specific policies to mitigate the gender disparity in decision making over access and control of production and household level resources. Third, unlike measures of education and age that are considered to be proxy/indirect measures of empowerment, the WEAI sub-indicators are direct measures of empowerment. Four, the WEAI accounts not just for women's empowerment but also the intra-household differences in empowerment levels between women and men.

While the WEAI has gained traction especially for identifying relationships between women's empowerment and nutritional outcomes (Cunningham et al., 2015; H. J. L. Malapit et al., 2015; Sraboni et al., 2014), its use has largely been restricted to household surveys implemented in the USAID's Feed the Future zones. The initial pilot locations included Bangladesh,^1^ Uganda^2^ and Guatemala.^3^ A look at the WEAI modules used in these pilots indicates that the individual-level questionnaires are identical across the three locations. In other words, there are no differences in the types of *activities* for which ownership and decision-making are assessed across countries. We find that the same set of activities are included across the surveys administered in Bangladesh, Uganda, Nepal, Malawi, Tajikistan, Ethiopia, Mozambique, Rwanda and Zambia.^4^ The use of the same list of activities for participation and decision-making suggests that agricultural practices are common across locations. This seems highly unlikely - the same set of activities/assets/sources of income or credit are less likely to be common across locations. In fact, a summary note^5^ from IFPRI compares WEAI results from Bangladesh, Ghana and Nepal to conclude that ‘patterns of disempowerment vary across contexts, and so should indicators and policy instruments.’ Such a statement is incomplete since while it advocates for the need for context-specific indicators the conclusion about differences in patterns of disempowerment is based on identical questionnaires across three different contexts. In the updated WEAI questionnaires^6^ while the questionnaire does recommend replacing examples with those relevant to a local context it stops short of saying anything about how these categories of activities were modified for the WEAI household surveys thus far.

Related to the issue of an identical set of activity categories is the fact that the WEAI stops short of addressing any associated changes in adequacy thresholds/cut-offs if the number of categories is in fact revised (over and above their constituent examples). The literature so far seems to assume that even if the activity-types are changed, the number of activities will remain the same (or greater), and therefore not require any modifications of adequacy thresholds. However, it is possible that the cutoffs used to identify women's empowerment in a given domain will need to be adapted in line with changes to the set of activities that constitute that particular domain.

And finally, there is no information in the public domain on WEAI analysis that looks at the sensitivity of the index to changes in thresholds used to identify empowerment. The original WEAI uses a threshold of 80% adequacy to identify women as being empowered in agriculture (Alkire et al., 2013) In other words, on a scale of 0–1, at a population-level woman in a given location are empowered in agriculture if the WEAI is greater than 0.8. According to IFPRI the 80% cutoff is neither too high (which might exclude too many women) nor too low (which might suggest it is easy to achieve empowerment/not much work needs to be done for improvement). There is no information in the public domain however on the results of the sensitivity analysis thus far. As part of our analysis, we accordingly investigate how the results from the WEAI change when the threshold used to identify disempowerment is modified.

For effective, targeted and efficient sectoral policy and program interventions, it is important to know which domains of empowerment matter for which region/context. Context plays an important role in achieving such an understanding. However, contextualizing an indicator comes at the cost of losing comparability across regions. Striking a balance between these two becomes challenging especially with a complex multidimensional indicator such as the WEAI. In this paper, we present results from our experience of adapting, implementing and analyzing the WEAI across multiple locations in India. In doing so we address three broad sets of limitations that, in our opinion, currently characterize the way the WEAI is being used by the international community: i) implementation based on underlying indicators that are not adapted to be context-specific, ii) adapting the way the index is constructed once the underlying activities and adequacy thresholds are modified and iii) analytical i.e. sensitivity and consistency analysis. Our primary objective is to construct the WEAI by adapting the metrics to an Indian agricultural context (WEAI_India). We do so by relying on context-specific, operational and well-defined indicators of access and ownership. In some instances, this is also accompanied by a change to the underlying cut-off that is used to identify women's empowerment in a given domain. We then test for how district-level empowerment statistics change as the threshold/cutoff for identifying empowerment changes. In our analysis, we vary the 80% threshold by considering two other formulations for the WEAI - a 40% and 60% adequacy in sub-indicators for identifying women's empowerment in agriculture. Lastly, we correlate the WEAI_India results with those from a reduced form of currently used indicator the A-WEAI (abbreviated-WEAI)^7^ to see how the results vary with the use of agriculture-specific indicators. This body of work builds on our previous experience of adapting the WEAI in the context of identifying differences in women's empowerment levels across different farming systems in India (Gupta, Pingali, and Pinstrup-Andersen 2017).

This study makes four key contributions. First, we provide an example of how the WEAI can be adapted based on challenges of implementing it as-is in an Indian context. This is in contrast to the way the WEAI has been used so far and is a first step in assessing how malleable the index is to site-specific characteristics. Recent discourse on measuring empowerment highlights the i) importance of using direct measures of empowerment rather than indirect measures that have been previously used ii) use of context and sector-specific measures (Malhotra, Schuler, and Boender 2002; Richardson, 2018) and iii) use of both universal and local indicators (Galiè et al., 2019). While our adaptation of the index is specific to India some of our adapted indices can be useful to other researchers as well. Second, by constructing the WEAI_India, we generate empirical evidence on the level of women's empowerment across four locations in the country and test for intra-country variations. Third, by carrying out sensitivity analysis, we contribute to the technical understanding of the WEAI. And finally, by comparing our results to the AWEAI we are able to identify aspects of empowerment that are picked up to different degrees by the two formulations.

We find that on average women in all four of our locations are disempowered in agriculture. The main drivers of women's disempowerment are absence of membership in agriculture-related Self-Help Groups (SHGs), ownership of land and control over income. Sensitivity analysis indicates that as the threshold is made loose, there is an improvement in empowerment status at the district level which is being driven by an associated change in the proportion of women who are identified as disempowered. Our consistency checks indicate that there are significant differences in the aggregate 5DE statistics between the existing tool (reduced_AWEAI) and our adaptation to India (WEAI_India) in each district.

The rest of this paper is structured as follows. In section 2 we describe the WEAI and discuss the challenges and methodology for adaptation of the index to an Indian context. Section 3 presents details on the data and methods. The results are presented in section 4. We conclude with recommendations in section 5.

## 2 Adapting the WEAI to an Indian context

Introduced in 2012, the WEAI measures assess women's access to resources and ability to make decisions in five domains of agriculture: 1) Production, 2) Resources, 3) Control over income, 4) Leadership and 5) Time use (Alkire et al., 2013). Since its introduction, the WEAI has been modified by a reduction in its scope. This was done because the original WEAI had been characterized as time and resource intensive to implement. In response, IFPRI has modified the original WEAI by reducing the number of sub-indicators from ten to seven: input in production decisions, ownership of assets, decisions on credit, control over income, group membership, workload and leisure. The three sub-indicators that were excluded were autonomy in decisions, public speaking and decisions related to the sale/purchase of various assets. This is also known as the Abbreviated WEAI or A-WEAI (H. J. Malapit et al., 2015).

The scope of the original WEAI was to identify a direct measure of women's empowerment with respect to the productive and economic activities *in agriculture*, and to be able to assess the gap in empowerment that women face in *agriculture* relative to men within the household (Arch and IFPRI 2012) The choice of expanding the scope of the index to non-agricultural domains can be dependent on the needs of the project – however our aim in this paper is to focus on the agricultural domains in particular. Accordingly, we adapt five of the seven AWEAI sub-indicators by changing the number and types of their constituent activities. For some sub-indicators (like input in production and group membership) we include more context-specific activities. For others (like control over income, credit and asset ownership) we restrict the set of activities to those related to agriculture alone, as opposed to including non-agricultural activities as well. Such modifications to sub-indicator activities may or may not be accompanied by a change in the adequacy threshold^8^ for a given sub-indicator. By making such changes, that essentially better reflect the agricultural context of our field-locations we arrive at, what we call, WEAI_India.

Our use of WEAI-India refers to an adaption of the WEAI that is relevant for our field locations – at the district level. By reorienting the focus on the index to agricultural activities and decision-making alone we are able to include a set of activities and assets that are relevant across our study locations. Taken together our set of activities do account for differences. For instance, the sub-indicator for input in production decisions includes ‘collection of forest produce’ as an activity – this however is predominantly applicable to our locations in Odisha, and not in UP and Bihar. It however does not affect the overall index construction since we are expanding the set of activities for that sub-indicator (and not reducing them), and therefore not changing the sub-indicator- level adequacy threshold. For other sub-indicators like asset ownership or credit we restrict the focus to direct, singular questions about access to resources that are – for example our focus on land ownership by women not only emphasizes the intrinsic and extrinsic importance of such ownership but is also based on the fact that more often it is the men who hold the title to the land. Our adaptation is based on a minimum set of common agricultural activities/assets that characterize the farming systems in our field locations. We acknowledge that this list of activities is open to modification based on differences in farming systems in other parts of the country. Having specified our scope and objectives, we describe the tool used to arrive at the activities as well as the adaptations the activities and thresholds in greater detail in section 3.3 and compare these modifications from the original AWEAI in Table 1.

**Table 1:** Differences in activities and adequacy thresholds between the WEAI_India & AWEAI.

Sub-indicator	AWEAI		WEAI_India		
	(Adequacy threshold) Activity	Weight	Activities	Weight	Any change in adequacy threshold?
Input in production decisions	*Has some input or feels can make a decision at least two activities* Food crop farming Cash crop farming Livestock raising Fisheries	1/5	*Has some input or feels can make a decision at least two activities* Which crops to plant Technology to adopt Sale of crops in market Buy/sell livestock Buy/Sell KG produce Collection of forest produce	1/5	No
Ownership of assets	*Adequate if self/joint owns at least two small assets or if households own one large asset* Agricultural land, large livestock, small livestock, fishing equipment, farm equipment (mechanized/non-mechanized), non-farm business equipment, house, large consumer durables, small consumer durables, cell phones, non- agricultural land, means of transportation	1/10	*Adequate if self/joint owns agricultural land* Agriculture land owned by women	1/10	Yes
Decisions on credit	*Adequate if self/joint makes decisions regarding credit and has at least one credit* NGO, formal lender, informal lender, friends and relatives, group-based MFI, informal group-based	1/10	*Adequate if self/joint can makes decisions regarding taking an agricultural loan* Taking a loan for agricultural activities	1/10	Yes
Control over income	*Adequate if there is at least one domain in which the individual has some input or feels can make decisions regarding wage employment and minor household expenditures.* Food crop farming, cash crop farming, livestock raising, non-farm activities, wage and salary employment, minor and major household expenditures & fishing	1/5	*Adequate if there is at least one domain in which the individual has some in controlling income* Income from sale of crops Income from sale of livestock Income from the collection of forest produce Income from ag- daily labor	1/5	Yes
Group membership	*Adequate if a woman is a member of at least one group* Agricultural/livestock/fisheries producer groups, water users group, forest users group, credit or microfinance group, mutual help/insurance group, Trade and business association group, civic group, other group	1/5	*Adequate if a woman is an SHG member and has joined SHG for:* SHG members who cited: Platform for doing collective livelihood Free seeds and samplings for homestead gardens Subsidized custom hiring of implements for agricultural activities Education about health, nutrition, education and WASH And Received training for agriculture activities, livestock activities and kitchen garden activities.	1/5	Yes
**Leisure**			Adequate if satisfied in leisure	1/5	No
**Workload**	Adequate if a woman works less than 10.5 h in previous 24 h (on primary activities – if required)	1/5	Seasonal, activity- specific time use		Alternate indicator, not included

## 3 Methods

### 3.1 Sites, sample and data collection

**Fig. 1:**
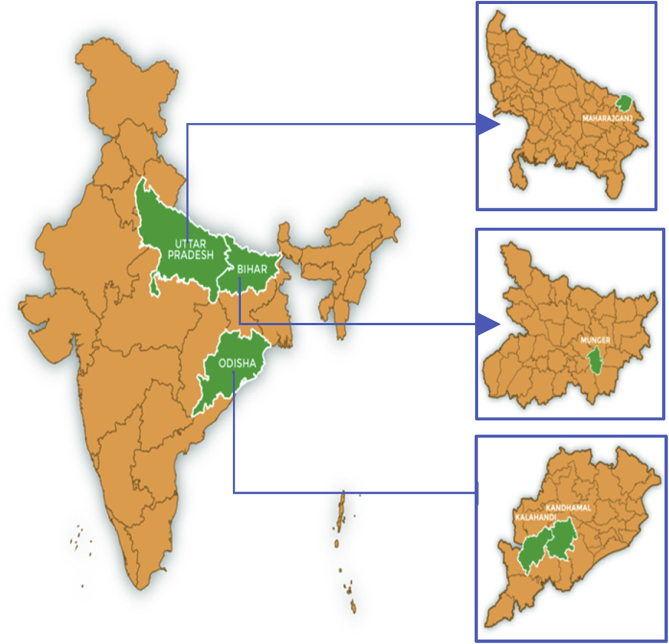
Field-locations: States and Districts.

We use primary data from a survey conducted in March-May 2017 as a part of Technical Assistance and Research for Indian Nutrition and Agriculture (TARINA) program in India. The TARINA program, led by the Tata- Cornell Institute for Agriculture and Nutrition (TCI) at Cornell University is a consortium of research and development organizations working on the design and promotion of nutrition-sensitive food systems in India. A total of 3600 households were surveyed as a part of the TARINA baseline survey in 2017. The survey was implemented in four districts: Munger (Bihar), Maharajganj (U.P), Kandhamal (Odisha) and Kalahandi (Odisha) – see Fig. 1.

The survey was designed using a two-stage sampling strategy. In the first stage, a total of 30 villages per district were selected based on population size and areas of implementation of the TARINA field-level partners. In the second stage, 30 households from each village were selected randomly from strata based on local caste groups. This resulted in a total sample of 3600 households across 120 villages. An index man and index woman were identified in each household. They were questioned about household demographics, agriculture and land use practices, dietary intake and factors influencing demand for food, empowerment and anthropometry. This study was approved by the Institutional Review Board at Cornell University. Informed consent was obtained verbally and recorded electronically.

### 3.2 Adapting the indicator-methods and variable construction

As mentioned earlier we adapt five sub-indicators of the AWEAI. In some cases, we restrict the set of activities (control over income, credit and asset ownership) and we make some more context specific (like input in production and group membership. The detailed list of activities included in the various agricultural domains was arrived at through a mixed methods approach. In 2016, a **diagnostics study** was undertaken by the TARINA team. The purpose of that study was to understand the status of different components of the local food systems. Within that there was a specific focus on assessing which agricultural activities women participate in (and by seasons) as well as women's participation in market-related activities like sale of crops etc. Moreover, the diagnostic study focused on the nature of different agricultural production systems. This allowed us, for instance, to identify the importance of collection and use of forest produce in our field locations in Odisha, and to identify that fisheries (an activity included in the original list of WEAI activities) was not relevant. Similarly, we realized the importance of focusing on women's engagement not just in production-related activities but also market-related activities like sale of crops/livestock etc. The diagnostic study was based on **focus group discussions** with different groups of respondents like male farmers, female farmers, landless farmers, and individuals segregated by caste groups. This was complemented with **in-depth interviews** with key informants as well as **transect walks** in select villages to see the nature of cropping systems, livestock engagement, home gardens and market-related activities across 3–4 villages in each district. Each FGD was conducted by three researchers from TCI and the partner organizations. The responses to FGD checklists were recorded manually and later transcribed/compiled at the end of each day. These were then used to identify the relevant themes/activities that eventually were incorporated in the women's empowerment module for the TARINA baseline survey. Following the diagnostics study the process of developing the survey questionnaire was initiated. This went through several rounds of review not just within the TCI research team but also in consultations with the TARINA **field partners**. Such a collaborative process ensured that we were able to maintain the context-specificity of the women's empowerment module in the household survey. Feedback was provided by the locally-recruited field team regarding the content, translation and the correct meaning of the questions-during the piloting of the questionnaire. All the feedback was incorporated in the final questionnaire which was administered to the respondents. The adaptations in terms of the inclusion of context specific activities, changes in thresholds made to each of the sub-indicator are explained in detail below:

#### 3.2.1 Adapting the inputs in production sub-indicator

The activities included in the input in production sub-indicator in the AWEAI are cash crop farming, food crop farming, livestock raising and fisheries. However, we found that i) fisheries were not a source of livelihood in our location and ii) that there were additional activities households engage in that were more relevant to their agricultural context. Accordingly, for this sub-indicator, we *expand* the list of activities to include location-specific participation in activities like crop cultivation, technology adoption, and marketing of kitchen garden produce, livestock/livestock produce and forest produce.

#### 3.2.2 Adapting the group membership sub-indicator

The AWEAI's group membership section focuses on whether or not the woman is a member of any group, with options being provided for different types of community-level groups. We found that the predominant aggregation model in our field sites was the Self- Help Group (SHG), model. Moreover, in focus group discussions with community members, we learned the specific types of activities/roles that the SHGs usually promoted. We used that information to reformulate the group membership questions to focus on the SHGs *and* account for their role in agriculture and nutrition in particular. For the group membership sub-indicator a woman is considered adequate if she is an SHG member AND the SHG acts as a platform for any one of the following: for doing collective livelihood/source of free seeds and samplings for homestead gardens/for access to subsidized custom hiring of implements for agricultural activities/for receiving education about health, nutrition, education and WASH/receiving training for agriculture activities, livestock activities and kitchen garden activities. These activities are either related to the provision of technical assistance related to agriculture and allied activities or related to the broader theme of health and nutrition.

#### 3.2.3 Adapting the ownership of assets sub-indicator

Both agricultural and non-agricultural assets are included in the calculation of the AWEAI's sub-indicator on asset ownership. We found it challenging to elicit responses related to ownership of most non-agricultural assets like consumer durables that can be considered household-level public goods and therefore for which ‘ownership’ is hard to determine. Even in the subset of agricultural assets, we find that the determination of ‘ownership’ of assets like small and large livestock is difficult. For instance, while women may be involved in the care of livestock, there is no way to distinguish their ‘ownership’ of said livestock from that of others in the household. Therefore, we argue that for an Indian context the most relevant asset is ownership of agricultural land, the property rights to which can be determined through a legal title/lease.

#### 3.2.4 Adapting the control over income sub-indicator

Similar to asset ownership, the AWEAI sub-indicator on control over income is calculated based on how much control the respondent feels he or she has over the use of income from a range of activities – both, agricultural and non-agricultural. While, non-agricultural activities are important for measuring empowerment in general, we include only income from agricultural activities such as the sale of crops, livestock, forest produce and income from daily agricultural wage labor. This is done to bring consistency by focusing only on agricultural domains when estimating empowerment levels in agriculture.

#### 3.2.5 Adapting the credit sub-indicator

A look at the credit sub-indicator the AWEAI indicates its focus on who applies for a loan in the household and whether or not a woman can make decisions about the use of that credit. While access to credit can be used as a proxy for access to resources of various kinds, we restricted the scope of that sub-indicator to access and use of agricultural credit alone, irrespective of the source of the loan. Once again, this is done for the same reason as described above.

Based on the modifications in the set of activities as described above we find that the adequacy threshold had to be adjusted for four of the AWEAI sub-indicators – ownership, credit, control over income and group membership. In the case of all of these sub-indicators the criteria for adequacy becomes tighter as the set of constituent activities is restricted. For instance, with respect to asset ownership, the requirements for adequacy become tighter since it is now based only on the ownership of one type of asset – agricultural land. The same is true for the credit sub-indicator which now focuses on a woman's ability to take decisions related to agricultural loans only. As for the control over income sub-indicator the adequacy threshold is now tighter since we account for a smaller set of agricultural activities.

### 3.3 Data analysis

Our construction of the WEAI_India follows the same methodology that has been developed for the WEAI (Alkire et al., 2013). The latter is the weighted sum of two sub-indices. The first is the five domains of empowerment sub-index (5DE) that focuses on the woman's adequacy in 80% of the sub-indicators, or four of the five domains. The second sub-index is the Gender Parity Index (GPI) that compares women's empowerment levels in agriculture to those of men in the same household, thereby accounting for equity in intra-household decision making and resource allocation. Taken together, a weighted average of the 5DE and GPI together makes up the WEAI at a population level, with the weights being 0.90 and 0.10 respectively. The WEAI ranges from 0 to 1 with a higher score indicating a higher level of empowerment.

For this paper, we compute the 5DE sub-index for the WEAI_India. The 5DE sub-index ranges from 0 to 1 with higher values indicating higher levels of empowerment. It is based on the proportion of disempowerment women (headcount ratio) and the average proportion of indicators in which disempowered women are disempowered (average inadequacy score). 5DE scores greater than 0.80 are used to characterize empowerment at the population level. We present aggregate and disaggregate statistics on the 5DE and its components for each district. Additionally we disaggregate the 5DE sub-index in order to arrive at the contribution (absolute and percentage) of each sub-indicator to overall disempowerment. For a detailed description of how the 5DE sub-index is constructed and decomposed to arrive at the contribution of individual sub-indicators to disempowerment, readers have referred to (Alkire et al., 2013).

While the TARINA baseline survey administered all seven sub-indicators to its sample, we restrict our analysis of the 5DE to six of them. We do not account for the workload in the 5DE computation. This is because it was computed using seasonal, activity-specific data that is outside the scope of this paper. Furthermore, we do not compute the GPI. While this means that our values for the WEAI_India are based on the 5DE sub-index alone, the fact that the GPI accounts for just 10% of the index weight leads us to believe that its exclusion will not influence the results significantly. To show the differences in the indices, we calculate the spearman rank order correlation between the reduced_AWEAI and the WEAI_India.

The original threshold of 20% implies that women who have inadequate decision-making or access to resources in 20% or less of sub-indicators would be categorized as disempowered in agriculture. This is the same as saying that their empowerment score is at least 0.80 (on a scale of 0–1). In our analysis we use two additional disempowerment thresholds – 40% and 60%. What this means is that we are increasing the range of sub-indicators in which women must be inadequate in order to qualify as being disempowered: at max 40% or at max 60% of the sub-indicators. That is the same as saying that we are making it ‘easier’ for women to be classified as ‘empowered’ since they now have to have adequacy in fewer sub-indicators. There are no set standards/guidelines for deciding on such a threshold for a given index. By using two alternate thresholds – 40% and 60% - we are able to assess change in statistics just below and just above the 50% threshold. We refer to the thresholds in terms of disempowerment in the rest of the manuscript. For each of these thresholds, we compute the 5DE sub-index separately for each district and test for group-level differences for the headcount ratio and average inadequacy scores between the different thresholds using ttests.

And finally, to test for **consistency** between our version of the WEAI_India with AWEAI we compare our results to a reduced form of the AWEAI and compare district 5DE results between the two formulations. The reduced_AWEAI focuses on a set of agricultural and non-agricultural activities that are a subset of the AWEAI. Appendix 1 compares the reduced_AWEAI to the original AWEAI and highlights the fact that the adequacy thresholds for the two do not change. We use the latter as a basis for using the reduced_AWEAI as a proxy for the AWEAI in our analysis. The empowerment score is constructed only if a woman's adequacy/inadequacy can be determined in all the sub-indicators. Therefore, if a respondent's adequacy level cannot be determined in even a single sub-indicator, that respondent is dropped from the calculations. Only if she participates in agriculture in the past 12 months, she is asked her input in decision making, control over income from agriculture and decisions regarding credit. Due to the lack of participation, her adequacy status cannot be determined in some of the sub-indicators- thus leading to a drop in the sample (See Appendix 3). Therefore, the method of construction of the index with a tighter set of activities included in the WEAI_India, the number of observations included for calculation of the index falls to around one-third of the total sample.

## 4 Results

Using the methods described in Section 3 and the adaptations made to the index (WEAI_India) we present findings on – i) the overall disempowerment levels across the study sites, ii) the contribution of each of the sub-domains to the overall disempowerment, iii) the changes in measures due to threshold differences and finally iv) the differences in measure using our adaptation versus the original index. Before we present findings related to the index, we describe the different characteristics of all the four study districts and contexts.

As represented in Table 2, the average age of women across districts is around 40 years. In Munger, Maharajganj and Kalahandi more than 70% of the women were illiterate. There is variation in the proportion of landless households across the four districts with there being as high as 54% such households in Munger and as low as 19% in Maharajganj. On average households in all districts except Kalahandi owned just under 2 acres of land. Production diversity, measured as the number of crops cultivated, is around 2 crops in Munger and Maharajganj and just 1 crop in the two districts of Odisha. Household market integration measured in terms of distance to local market from the village also varies across districts. In Odisha, the market is 2–5 kms away from at least two-thirds of the villages while in Munger and Maharajganj one-fifth of the villages had the local market present in the village itself.

**Table 2:** Summary statistics across the four study locations.

	Bihar	U.P.	Odisha	
	Munger	Maharajganj	Kandhamal	Kalahandi
Age of women	38.1	36.0	40.2	37.6
Education				
Illiterate	77.6%	74.6%	48.5%	73.8%
School	17.7%	17.7%	42.4%	24.9%
Intermediate	3.3%	5.4%	7.3%	1.2%
College	1.2%	2.2%	1.9%	0.1%
Vocational	0.2%	0.1%	0.0%	0.0%
Landless	53.7%	19.5%	46.3%	31.1%
Land ownership (acre)	1.8	1.6	1.9	2.7
Caste				
OBC	42.2%	75.5%	12.3%	28.4%
SC	23.4%	23.3%	30.8%	27.1%
ST	30.1%	0.8%	55.8%	43.7%
General	4.2%	0.5%	1.1%	0.8%
Number of crops grown	2.1	2.6	1.3	1.3
Market distance				
In village	26.7%	20.1%	0.0%	30.0%
Within 2 k.m.	40.0%	26.8%	6.5%	10.1%
Between 2-5 k.m.	33.3%	53.2%	93.5%	59.9%

### 4.1 Women's empowerment & key drivers of disempowerment based on WEAI_India

Table 3 describes district-level results for women's empowerment in agriculture based on the WEAI_India. The 5DE scores in our sample indicate that women in all four districts are disempowered (5DE < 0.80). Across districts, at least 80% of women are disempowered, with the highest proportion being in Maharajganj (95% women). The average inadequacy scores are lowest in Munger, and similar for the other three districts. While women on average have inadequacy in 40% of the domains in Munger, for UP and Bihar this proportion rises to slightly more than half.

**Table 3:** Women's empowerment in agriculture across districts (the five domains of empowerment sub-index (5DE) for WEAI_India).

Indices	Bihar	U.P.	Odisha	
	Munger	Maharajganj	Kandhamal	Kalahandi
Disempowered Headcount (H)	82.7%	94.5%	83.5%	85.4%
Average Inadequacy Score (A)	40.2%	51.3%	53.2%	51.2%
Disempowerment Index (M0 = H x A)	33.2%	48.5%	44.4%	43.7%
Five domains of empowerment sub-index (5DE) (1-M0)	66.8%	51.5%	55.6%	56.3%
No. of observations used	289	255	321	356
Total observations	900	900	900	900
% of Data used	32.1%	28.3%	35.7%	39.6%

**Fig. 2:**
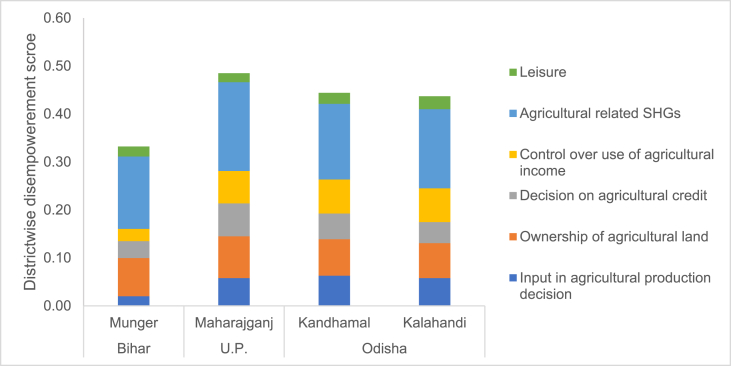
Absolute contribution of sub-indicators to women's disempowerment.

Next, upon disaggregating the WEAI_India we see that in absolute terms the main driver of women's disempowerment is the absence of group membership - it accounts for approximately 40% of the burden of disempowerment across districts. This is followed by women's lack of ownership of agricultural land that accounts for one-fifth of the overall disempowerment in agriculture in all four districts. Women's control over agricultural income and input in agricultural production activities is relatively lower (contribution to disempowerment is higher) in Odisha as compared to UP and Bihar. Leisure accounts for less than 10% of the overall disempowerment across districts. The absolute contribution of the sub-indicator on women's input in agricultural production decisions is lowest in Munger. Fig. 2 summarizes the absolute contribution of each sub-indicator to overall disempowerment of women in each district.

### 4.2 Sensitivity analysis

Table 4 presents district disempowered headcount, average inadequacy score and 5DE scores for the WEAI_India using three thresholds: 20%, 40% and 60% and indicates if differences within districts across thresholds are significant or not. We find that as the threshold for identifying disempowerment increases there is an increase in 5DE scores. Relative to the original threshold of 20% for identifying disempowerment, an increase to 40% and 60% results in average district 5DE scores that indicate women are empowered in each of the four districts. This improvement in empowerment status at the district level is being driven by an associated change in the proportion of women who are identified as disempowered. We find that there is a significant decline in the proportion of disempowered women in each of the four districts. At the 20% threshold at least 80% women in each district were disempowered. This reduces significantly by half (in UP and Odisha) or more (in Bihar) as the threshold is relaxed to 40%. At the 60% cutoff, we find less than 10% of the women in Munger qualify as disempowered while those in the remaining three districts are about double of this.

**Table 4:** Sensitivity analysis: changes in disempowerment measures using different cutoffs for identifying as empowered or not.

	WEAI_India thresholds for disempowerment		
	**20%***(Disempowered:* *5DE < 0.80)*	**40%***(Disempowered: 5DE < 0.60)*	**60%***(Disempowered: 5DE < 0.40)*
**Munger**			
Disempowered headcount*	82.7%	19.4%	6.2%
Average inadequacy	40.2%	61.8%	77.2%
5DE	66.8%	88.0%	95.2%
**Maharajganj**			
Disempowered headcount*	94.5%	47.5%	20.4%
Average inadequacy	51.3%	67.0%	80.6%
5DE	51.5%	68.2%	83.6%
**Kandhamal**			
Disempowered headcount*	83.5%	46.1%	24.6%
Average inadequacy	53.2%	69.1%	80.5%
5DE	55.6%	68.2%	80.2%
**Kalahandi**			
Disempowered headcount*	85.4%	42.7%	24.4%
Average inadequacy	51.2%	70.4%	82.4%
5DE	56.3%	69.9%	79.9%

Note: *Test of difference in proportions for the disempowered headcount between 20 and 40% thresholds, and between 20% and 60% thresholds are significant at 1% level for all districts.

The decline in disempowered headcount taken together with the increase in the average inadequacy implies that even though fewer women are disempowered, the average proportion of sub-indicators they are disempowered in is now higher. We find that the magnitude of the decline in the disempowered headcount is greater than the magnitude of increase in the average inadequacy scores across districts. It is this difference that contributes to the higher 5DE scores as the thresholds are varied.

### 4.3 Consistency analysis

In this section, we compare results from the WEAI_India to a reduced form of the AWEAI (i.e. reduced_AWEAI), using the original threshold of 20% to identify disempowerment. We find that there are significant differences in the aggregate 5DE statistics between the reduced_AWEAI and WEAI_India in each district (Table 5). For one, after restricting the scope of the WEAI to agriculture only (i.e. WEAI_India), all districts have a 5DE score that is well below the 0.80 thresholds. This is unlike the reduced_AWEAI results where women in Munger were empowered in agriculture while those in Maharajganj, Kandhamal and Kalahandi were not. A look at the components of the 5DE sub-index in Table 5 suggests that relative to the WEAI_India, the reduced_AWEAI underestimates the proportion of disempowered women in each district. Across districts, at least 82% of women are disempowered as per the WEAI_India whereas this figure is as low as 24% based on the reduced_AWEAI. This is likely to being driven by the fact that the adequacy thresholds for the WEAI_India are tighter. In addition, we calculate the spearman rank order correlation coefficient for the ordinal empowerment variable using the reduced_AWEAI and the WEAI_India at the original cut-off of 1 percent 20 percent and we find that the coefficient is 0.21 and 0.38 respectively. Both these coefficients are significant at 1 percent significance level reflecting a weak correlation between the two indices supporting the case for a more context specific indicator.

**Table 5:** Comparison of results: WEAI_India vs reduced_AWEAI.

Indices	Bihar		U.P.		Odisha			
	Munger		Maharajganj		Kandhamal		Kalahandi	
	Reduced_AWEAI	WEAI_India	Reduced_AWEAI	WEAI_India	Reduced_AWEAI	WEAI_India	Reduced_AWEAI	WEAI_India
Disempowered Headcount (H)^a^	24.4%	82.7%	63.8%	94.5%	53.0%	83.5%	46.2%	85.4%
Average Inadequacy Score (A)^b^	41.5%	40.2%	46.5%	51.3%	51.3%	53.2%	52.5%	51.2%
5DE Index (1-M0)	89.9%	66.8%	70.3%	51.5%	72.8%	55.6%	75.7%	56.3%

a Test of difference in proportions between AWEAI and RWEAI are significant at 1% level for all districts.

b T-test analysis for the difference in means between AWEAI and RWEAI are significant at 1% level for all districts.

**Fig. 3:**
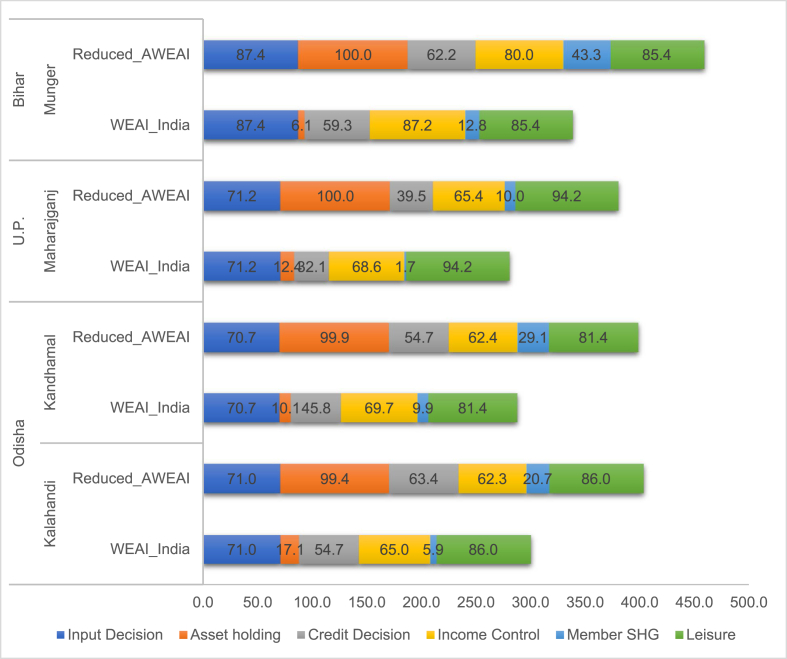
Percentage of women who are empowered across sub-indicators for the WEAI_India and reduced_AWEAI in each district.

Since we know that there is a significant difference in the disempowered headcount based on the WEAI_India and reduced_AWEAI we disaggregate the 5DE scores from each of those formulations to identify differences at the level of sub-indicators. Fig. 3 shows how the proportion of women who are empowered drops significantly when we move from the reduced_AWEAI to WEAI_India for each of those sub-indicators that we adapted to an Indian context and for which adequacy thresholds were also modified-asset ownership, credit decisions, and group membership. This difference holds true across districts. The biggest absolute reduction in women who are empowered is seen with respect to *asset ownership.* While nearly all the women are empowered in this sub-indicator based on the reduced_AWEAI, the proportion falls to around 10% on average across districts when the WEAI_India is used, i.e. once assets are restricted to agricultural land only. The next big decline in the proportion of women who are empowered is seen with respect to the group membership sub-indicator. We note that once the criteria for group membership is restricted to agriculture-focused SHGs, then 10% or fewer women are empowered in this sub-indicator. The WEAI_India identifies marginally lower and higher proportions of women who have access to credit and control over income respectively, as compared to the reduced_AWEAI. This result for the control over income sub-indicator is interesting since even with a smaller set of agricultural activities the WEAI_India identifies a larger proportion of women as being empowered. This suggests that the specific agricultural activities we include in this sub-indicator are better able to pick up women's ability to control income as compared to those included in the reduced_AWEAI.

Even though we modify the input in production sub-indicator since there is no change in its adequacy threshold, we find that there is no difference in the proportion of women who are empowered in it between the two formulations. The empowered headcount is also identical for the leisure sub-indicator as it remains unchanged in our calculations.

## 5 Discussion and conclusion

The need for direct, context and sector-specific measures of women's empowerment has been highlighted in the recent discourse. The women's empowerment in agriculture index (WEAI) and its subsequent variants such as A-WEAI have taken the lead in this direction in an agricultural context. While the WEAI and the A-WEAI are extremely useful indices and give the ability for researchers as a direct, domain-based measure; the need to contextualize the tools to specific contexts and programs is important. In this paper, we contribute to this discourse in detail. Based on our experience of implementing the index across four locations in India, we demonstrate how we adapted the WEAI to site-specific, well-defined indicators of women's role in agriculture. We find that when we attempt to do such an exercise for an Indian agricultural context, we end up with a smaller set of relevant agricultural activities often accompanied by tighter adequacy requirements as compared to the AWEAI. We conclude that when a narrower, well-defined a set of activities are included in the sub-indicators then, for the most part, there is a reduction in the proportion of women who are empowered in those sub-indicators.

Furthermore the extent to which adequacy thresholds for a given sub-indicator are modified influences the proportion of women who are identified as empowered or not. This in turn will influence the aggregate 5DE scores for a given region. And finally, the interpretation of the aggregate 5DE scores depends on the threshold used for proportion of sub-indicators in which women need to be empowered. Through sensitivity analysis, we show that reducing the cut-off significantly increases the proportion of empowered women in each district.

Our work is limited in that we do not account for the time-use sub-indicator in our construction of the WEAI_India. This is mainly because we followed a different methodology to account for women's time use in agriculture. That was based on time spent in disaggregated agricultural activities in multiple cropping seasons. Such data cannot be reconciled with a one-time measure of their engagement in other agricultural domains (as is the case in the AWEAI) and has therefore been excluded from the analysis.

In this paper, we present a methodology for how the WEAI can be adapted to suit contextual and operational requirements. With this approach, we may still account for the same construction methodology; we can identify the specific components of sub-indicators that are restricting women's empowerment. Accordingly, any policy that is formulated to address them will be based on a site-specific understanding of the activities that comprise the sub-indicator as opposed to a standardized list of activities/components. For instance, we find that the drivers of women's disempowerment based on the WEAI_India are group membership, asset ownership and decision making related to agricultural credit. These are like those identified by (Gupta, Pingali, and Pinstrup-Andersen 2017) for Maharashtra, India. The cross-country WEAI baselines indicate that credit and group membership are also areas that contribute the most to women's disempowerment. What sets the interpretation of our results apart however, is the fact that we know precisely what aspect of these sub-indicators is driving women's disempowerment. For instance, in this case, it is women's lack of access to and decisions related to agricultural SHGs, land and credit. We hope that this exercise will prove useful to other researchers in adapting the index to their contexts and requirements. While building up an evidence base for the WEAI is important, we think that such measurement makes it richer by making its context and site-specific. This will not only help in identifying the specific domains of disempowerment but also ensure a targeted and efficient program/policy interventions.

## Funding

This work was supported by the Bill & Melinda Gates Foundation, Seattle, WA [# OPP1137807]. The funding agency was not involved in the study design; collection, analysis and interpretation of the data; in the writing and preparation of the report; and in the decision to submit the article for publication.

## Declaration of interest

None.

## Figures and Tables

**Table:** 

Sub-indicator	Activities included in		
	AWEAI	Reduced_AWEAI	The difference in the threshold for reduced_AWEAI as compared to AWEAI
Input in production decisions	*Has some input or feels can make decision at least two activities* Food crop farming Cash crop farming Livestock raising Fisheries	Has some input or feels can make decision at least two activities Which crops to plant Technology to adopt Sale of crops in the market Buy/sell livestock Buy/Sell KG produce Collection of forest produce	No
Ownership of assets	*Adequate if self/joint owns at least two small assets or if households own one large asset* Agricultural land, large livestock, small livestock, fishing equipment, farm equipment (mechanized/non-mechanized), non-farm business equipment, house, large consumer durables, small consumer durables, cell phones, non- agricultural land, means of transportation	Agricultural land owned by women, house, phone, Large livestock, Small livestock	No
Decisions on credit	*Adequate if self/joint makes decisions regarding credit and has at least one credit* NGO, formal lender, informal lender, friends and relatives, group-based MFI, informal group-based.	Taking a loan for Agricultural activities Taking a loan for Non-Agricultural activities	No
Control over income	*Adequate if there is at least one domain in which the individual has some input or feels can make decisions regarding wage employment and minor household expenditures.* Food crop farming, cash crop farming, livestock raising, non-farm activities, wage and salary employment, minor and major hh expenditures fishing.	Income from sale of crops Income from sale of livestock Income from the collection of forest produce Income from ag- daily labor Income from non-farm labor	No
Group membership	*Adequate if a woman is a member of at least one group* Agricultural/livestock/fisheries producer groups, water users group, forest users group, credit or microfinance group, mutual help/insurance group, Trade and business association group, civic group, other group	*Adequate if a woman is a member of a Self Help Group (SHG)*	Yes
**Workload**		Seasonal, activity- specific. Analyzed separately from the WEAI analysis (see section 2.4).	NA
**Leisure**		Same as AWEAI	NA

**Table:** 

Indices	Bihar	U.P.	Odisha	
	Munger	Maharajganj	Kandhamal	Kalahandi
Disempowered Headcount (H)	7.6%	31.3%	30.0%	26.8%
Empowered Headcount (1-H)	92.4%	68.7%	70.0%	73.2%
Average Inadequacy Score (A)	60.7%	60.4%	63.5%	64.4%
Average Adequacy Score (1- A)	39.3%	39.6%	36.5%	35.6%
Disempowerment Index (M0 = H x A)	4.6%	18.9%	19.0%	17.3%
5DE Index (1-M0)	95.4%	81.1%	81.0%	82.7%
No. of observations used	356	307	387	463
Total observations	900	900	900	900
% of Data used	39.6%	34.1%	43.0%	51.4%
Disempowered Headcount (H)	3.9%	14.0%	17.6%	14.7%
Empowered Headcount (1-H)	96.1%	86.0%	82.4%	85.3%
Average Inadequacy Score (A)	70.0%	70.9%	72.5%	73.7%
Average Adequacy Score (1- A)	30.0%	29.1%	27.5%	26.3%
Disempowerment Index (M0 = H x A)	2.8%	9.9%	12.7%	10.8%
5DE Index (1-M0)	97.2%	90.1%	87.3%	89.2%
No. of observations used	356	307	387	463
Total observations	900	900	900	900
% of Data used	39.6%	34.1%	43.0%	51.4%

Reduced_AWEAI results using 40% threshold.

Reduced_AWEAI results using 60% threshold.

**Table:** 

A-WEAI_Reduced - missing numbers by domain					
	Munger	Maharajganj	Kandhamal	Kalahandi	Overall
Input in decisions	456	515	450	337	1758
Asset Ownership	0	0	0	0	0
Decisions on credit	337	262	262	209	1070
Control over income	215	230	197	118	760
Group membership	0	0	0	3	3
Leisure	1	1	0	0	2
Overall	544	593	513	437	2087
**WEAI_India - missing numbers by domain**					
Input in decisions	456	515	450	337	1758
Asset Ownership	0	0	0	3	3
Decisions on credit	605	635	575	529	2344
Control over income	470	556	461	349	1836
Group membership	0	0	0	0	0
Leisure	1	1	0	0	2
Overall	611	645	579	544	2379

## Footnotes

1 Pilot questionnaire available at https://dataverse.harvard.edu/file.xhtml?fileId=2425427&version=3.1.

2 Pilot questionnaire available at https://dataverse.harvard.edu/file.xhtml?fileId=2425635&version=3.1.

3 individual questionnaire is available only in Spanish: https://dataverse.harvard.edu/file.xhtml?fileId=2425626&version=4.4.

4 All the datasets and questionnaires can be found at https://data.usaid.gov/Agriculture/Feed-The-Future-Zambia-Baseline-Population-Based-S/4vjz-rqba.

5 https://dataverse.harvard.edu/file.xhtml?persistentId=doi:10.7910/DVN/DXMARV/EKCS8W.

6 Available at https://weai.ifpri.info/weai-resource-center/guides-and-instruments/.

7 From now on we refer to this indicator as the reduced_AWEAI.

8 More specifically, it is a *reduction* in the relevant set of activities that can be problematic for using the adequacy thresholds specified in the WEAI for each sub-indicator. For instance, let's say the input in production decisions focuses on the same set of three activities for locations A and B – buying seeds, selling crop and buying/selling of livestock. Now assume that all three are relevant for location A but only two of the three are relevant for location B. If all three activities are included in the survey, the adequacy requirement for this sub-indicator is that the woman should participate in at least 2 activities. If however we were to restrict the activities for location B to only the two that are relevant then the original adequacy requirement will effectively mean that the woman participates in *all* 2 out of 2 activities in location B.
